# Sensitive Real-Time PCR Detection of Pathogenic *Leptospira spp*. and a Comparison of Nucleic Acid Amplification Methods for the Diagnosis of Leptospirosis

**DOI:** 10.1371/journal.pone.0112356

**Published:** 2014-11-07

**Authors:** Jesse J. Waggoner, Ilana Balassiano, Janaki Abeynayake, Malaya K. Sahoo, Alisha Mohamed-Hadley, Yuanyuan Liu, Juliana Magalhães Vital-Brazil, Benjamin A. Pinsky

**Affiliations:** 1 Department of Medicine, Division of Infectious Diseases and Geographic Medicine, Stanford University School of Medicine, Stanford, California, United States of America; 2 Laboratório de Zoonoses Bacterianas, Centro de Referência Nacional para Leptospirose, Coleção de Leptospira, WHO/PAHO Centro Colaborador para Leptospirose, Instituto Oswaldo Cruz, Fiocruz, Rio de Janeiro, Brazil; 3 Department of Pathology, Stanford University School of Medicine, Stanford, California, United States of America; Naval Research Laboratory, United States of America

## Abstract

**Background:**

Bacteria of the genus *Leptospira*, the causative agents of leptospirosis, are categorized into pathogenic and non-pathogenic species. However, the benefit of using a clinical diagnostic that is specific for pathogenic species remains unclear. In this study, we present the development of a real-time PCR (rtPCR) for the detection of pathogenic *Leptospira* (the pathogenic rtPCR), and we perform a comparison of the pathogenic rtPCR with a published assay that detects all *Leptospira* species [the undifferentiated febrile illness (UFI) assay] and a reference 16S *Leptospira* rtPCR, which was originally designed to detect pathogenic species.

**Methodology/Principal Findings:**

For the pathogenic rtPCR, a new hydrolysis probe was designed for use with primers from the UFI assay, which targets the 16S gene. The pathogenic rtPCR detected *Leptospira* DNA in 37/37 cultured isolates from 5 pathogenic and one intermediate species. Two strains of the non-pathogenic *L. biflexa* produced no signal. Clinical samples from 65 patients with suspected leptospirosis were then tested using the pathogenic rtPCR and a reference *Leptospira* 16S rtPCR. All 65 samples had tested positive for *Leptospira* using the UFI assay; 62 (95.4%) samples tested positive using the pathogenic rtPCR (p = 0.24). Only 24 (36.9%) samples tested positive in the reference 16S rtPCR (p<0.0001 for comparison with the pathogenic rtPCR and UFI assays). Amplicon sequencing confirmed the detection of pathogenic *Leptospira* species in 49/50 cases, including 3 cases that were only detected using the UFI assay.

**Conclusions/Significance:**

The pathogenic rtPCR displayed similar sensitivity to the UFI assay when testing clinical specimens with no difference in specificity. Both assays proved significantly more sensitive than a real-time molecular test used for comparison. Future studies are needed to investigate the clinical and epidemiologic significance of more sensitive *Leptospira* detection using these tests.

## Introduction

Leptospirosis is a potentially fatal systemic illness resulting from infection with spirochetes of the genus *Leptospira* [1], [2]. Humans are accidental hosts and typically acquire the infection from direct contact with water contaminated with the urine of small mammals [1]–[3]. A wide range of clinical manifestations can occur following human infection with *Leptospira*, spanning asymptomatic infection to severe disease, multi-system organ failure and death [1], [2], [4]–[6]. It is estimated that 873,000 severe infections occur annually, with 49,000 deaths [3]. However, the non-specific disease presentation and limitations in available diagnostics for *Leptospira* likely render these disease estimates inaccurate [1], [2], [7], [8]. While debate exists regarding the efficacy of antibiotics in severe leptospirosis, accurate and early diagnosis may improve patient outcomes by allowing for the timely administration of antibiotic therapy [9], [10].

Currently, the reference standards for the diagnosis of leptospirosis remain bacterial culture and serological microscopic agglutination testing (MAT) [3], [11]. These tests are both resource intensive and cannot provide results in a clinically meaningful timeframe. The culture of *Leptospira* requires up to four weeks for results and is insensitive compared to other techniques [1], [2], [11], [12]. MAT requires the maintenance of a local reference panel of live bacterial cultures for assay performance and the use of acute and convalescent samples to provide a confirmed diagnosis [2], [3], [11], [13]. Point-of-care serological diagnostics have been developed for *Leptospira* and allow earlier detection of IgM than MAT [3], [14]. Such assays are less clinically sensitive than MAT, however, and only provide a presumptive diagnosis [14].

Many different molecular diagnostics for leptospirosis have been developed [11], [15]–[25]. These tests can offer sensitive *Leptospira* detection while also providing a definitive diagnosis in acute disease [3], [13], [20], [26], [27]. Many of these tests have been designed to specifically detect pathogenic *Leptospira* species [8], [21], [22], [28]. However, the use of such assays for testing clinical specimens has not consistently resulted in improved diagnostic accuracy [2], [8], [19], [20]. This may have resulted from the decreased clinical sensitivity of specific assays [8] combined with the absence of detection of non-pathogenic species in sterile specimens [20]. Recently, our group reported the development of a real-time PCR (rtPCR) for the detection of all *Leptospira* species (pathogenic and non-pathogenic), which is included in a multiplex assay for the diagnosis of dengue, leptospirosis, and malaria [termed the undifferentiated febrile illness (UFI) assay]. The UFI assay proved significantly more sensitive than conventional PCRs for the detection of *flaB* and *lipL41* [29]. However, the use of conventional PCRs for comparison may have accounted for the increased sensitivity of the UFI assay, which, to date, has not been evaluated against another real-time molecular diagnostic. Furthermore, it was unclear if similar results could be obtained using an assay specific for pathogenic *Leptospira* species.

In the current study, we report the development and analytical characterization of an rtPCR for the detection of pathogenic *Leptospira* species (referred to as the pathogenic rtPCR). The pathogenic rtPCR combines the *Leptospira* primers from the UFI assay with a new hydrolysis probe that targets sequence found in pathogenic *Leptospira* species. This assay was performed as a monoplex reaction, which differs from the multiplex, internally-controlled design of the UFI assay. Using 65 clinical samples from suspected leptospirosis cases in Brazil, we then performed a comparison of the UFI assay and pathogenic rtPCR with a reference16S rtPCR [22].

## Methods

### Ethics

The Stanford University IRB waived review of this study. All samples were pre-collected as part of routine clinical care and de-identified.

### Assay Design

**Figure 1:**
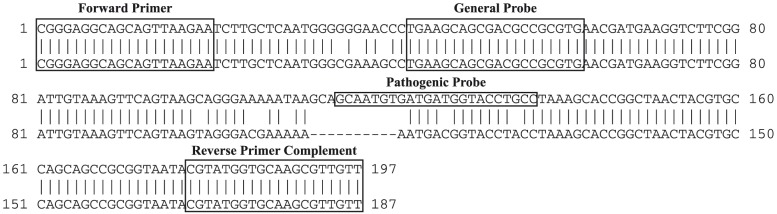
BLAST alignment for the targeted 16S rRNA gene consensus sequences from pathogenic (top) and non-pathogenic (bottom) strains of *Leptospira*. The forward primer sequence and the complement of the reverse primer sequence used in the pathogenic rtPCR and UFI assay are labeled, as are the general (UFI assay) and pathogenic probe sequences.

The pathogenic rtPCR utilizes primers that were developed for the UFI assay. These primers target a region of the *Leptospira* 16S rRNA (rrs) gene (Figure 1), and their design has been described previously [29]. To design the pathogenic probe, sequences that matched conserved regions of available *L. biflexa* sequences and differed from *L. interrogans* sequences were removed from an alignment of 704 sequences of the *Leptospira* 16S rRNA gene. These were re-aligned as non-pathogenic species using MegAlign software (DNASTAR, Madison, WI). This alignment included all available sequences for *L. biflexa* (n = 12) and the following 22 sequences: *L. meyeri* (n = 15); *L. wolbachii* (n = 2); *L. vanthielii* (n = 2); *L. idonii*, *L. terpstrae*, and *L. yanagawae* (1 each; accession numbers available on request). The remaining 670 sequences from pathogenic species were re-aligned. These pathogenic and non-pathogenic consensus sequences were then aligned using BLAST to identify targets for a pathogen specific probe (Figure 1). The pathogenic probe (5′ – Cal Fluor 560 – GCRATGTGATGATGGTACCTGCCT – BHQ-1 – 3′) was designed using Primer-BLAST [30].

### Assay Performance and Reference PCRs

Pathogenic rtPCR reactions were performed on the Rotor-Gene Q instrument (Qiagen, Germantown, MD) using 25 µL reaction volumes and the SuperScript III Platinum One-Step qRT-PCR kit (Invitrogen, Carlsbad, CA). Each reaction contained 5 µL of nucleic acid template; forward and reverse primers as well as the probe were used at final concentrations of 400 nM. Cycling conditions were identical to conditions described for the UFI assay [29]. During analysis, the first five cycles were cropped to improve baseline normalization. Results were evaluated on the linear scale with slope correction and a threshold of 0.05. A positive result was considered any exponential curve with a cycle threshold (C_T_) prior to cycle 45.

The 16S rtPCR developed by Smythe, et al., was used as reference for the testing of cultured *Leptospira* strains and clinical samples [22]. This assay was performed on the Rotor-Gene Q instrument using 25 µL reaction volumes of the TaqMan Universal PCR Master Mix (Life Technologies, Grand Island, NY). Each reaction contained 5 µL of nucleic acid template. Primer and probe concentrations were used according to Thaipadunpanit, et al [8]. Cycling conditions were the following: 95°C for 10 min and 45 cycles of 95°C for 15 sec and 60°C for 60 sec. Detection was performed in the green channel at 60°C; the gain was set at 10 following optimization. During analysis, the first five cycles were cropped; results were evaluated on the linear scale with slope correction and a threshold of 0.05. A positive result was considered any exponential curve with a C_T_ prior to cycle 45. A no-template control was included on each run of the UFI assay, pathogenic, and reference 16S rtPCRs. No signal was observed from the no-template control on any run.

Clinical samples were originally sent to the Laboratório de Zoonoses Bacterianas, Instituto Oswaldo Cruz, Fundação Oswaldo Cruz (Fiocruz) and tested with PCRs for *flaB* and *lipL41* as described [16], [25], [29].

### Control Nucleic Acids and Reference Material

The analytical validation of the pathogenic rtPCR was performed using quantitated plasmid DNA. *Leptospira interrogans* serovar Copenhageni, strain Fiocruz L1-130 (ATCC Number BAA-1198D-5; ATCC, Manassas, VA) genomic DNA was used during plasmid production, as previously described [29]. This is referred to as the *Leptospira* reference strain. Extracted genomic DNA from 39 cultured *Leptospira* isolates was tested using the pathogenic and 16S reference rtPCRs. These included strains from 7 species and 23 different serovars of *Leptospira* (Table 1).

**Table 1:** Reference *Leptospira* isolates tested with the pathogenic and reference 16S rtPCRs.

Leptospires Obtained from the *Leptospira* Collection (CLEP) – Fiocruz (Rio de Janeiro, Brazil)			
Species	Serovar	Strain	CLEP Code
*L. biflexa*	Semaranga	Patoc 1	00015
*L. biflexa*	Andamana	CH11	00021
*L. borgpetersenii*	Tarassovi	Perepelitsin	00016
*L. borgpetersenii*	Javanica	Veldrat Batavia 46	00010
*L. borgpetersenii*	Castellonis	Castellon 3	00008
*L. fainei*	Hurstbridge	But 6	00026
*L. interrogans*	Icterohaemorrhagiae	RGA	00001
*L. interrogans*	Copenhageni	M20	00002
*L. interrogans*	Canicola	Hond Utrecht IV	00003
*L. interrogans*	Pomona	Pomona	00005
*L. interrogans*	Australis	Ballico	00006
*L. interrogans*	Autumnalis	Akiyami A	00017
*L. interrogans*	Pyrogenes	Salinem	00012
*L. interrogans*	Lai	Lai	00028
*L. kirshneri*	Grippotyphosa	Moskva V	00004
*L. kirshneri*	Mozdok	5621	00091
*L. noguchii*	Panama	CZ214K	00011
*L. weilii*	Vughia	LT 89–68	00040

Leptospires Obtained from Queensland Health (Queensland, Australia)	
Species	Serovar
*L. borgpetersenii*	Arborea
*L. borgpetersenii*	Hardjo (Bovis)
*L. borgpetersenii*	Tarassovi
*L. interrogans*	Australis
*L. interrogans*	Bindjei
*L. interrogans*	Broomi
*L. interrogans*	Canicola
*L. interrogans*	Copenhageni
*L. interrogans*	Hardjo
*L. interrogans*	Kremastos
*L. interrogans*	Mankarso
*L. interrogans*	Medanensis
*L. interrogans*	Pomona
*L. interrogans*	Robinsoni
*L. interrogans*	Szwajizak
*L. interrogans*	Valbuzzi
*L. interrogans*	Zanoni
*L. kirschneri*	Bulgarica
*L. kirschneri*	Grippotyphosa
*L. weilii*	Celledoni
*L. weilii*	Topaz

### Analytical Characterization

The pathogenic rtPCR was analytically characterized according to published recommendations [31]. To establish the dynamic range of the pathogenic rtPCR, four replicates of serial 10-fold dilutions of plasmid DNA from 7.0 log_10_ copies/µL to 1 copy/µL were tested in a single run. The dynamic range of the reference 16S rtPCR was evaluated by testing four replicates of serial 10-fold dilutions of genomic DNA from the *Leptospira* reference strain from 5.0 to 1.0 log_10_ copies/µL. The concentration, in copies/µL, of the *Leptospira* reference strain was calculated from the standard curve generated during the pathogenic rtPCR dynamic range evaluation. The dynamic range was established for each assay by fitting a best-fit line to the data by regression analysis and included the range where the R^2^ value for this line was ≥0.99.

To establish the lower limit of 95% detection (95% LLOD) for the pathogenic rtPCR, ten replicates of four, two-fold dilutions were tested on a single run. The 95% LLOD was then calculated using probit analysis. The dilutions began at a concentration 2-fold higher than the lowest concentration at which all replicates were detectable during the dynamic range study.

The specificity of the pathogenic rtPCR was evaluated by testing serum samples from 99 Nicaraguan dengue cases. These samples have been described previously [32]. In addition, specificity was evaluated by testing extracted DNA from clinical strains of *Staphylococcus aureus* and coagulase-negative *Staphylococcus* (three strains each); and cultured strains of *Salmonella enterica* subsp. *arizonae*, *S. enterica* serovar Typhi, *Treponema denticola* (ATCC strain 35405), and *Borrelia burgdorferi* strain B31 (ATCC number 35210).

### Clinical Samples

Archived samples (63 serum, 2 plasma) collected from 65 patients in Brazil with suspected leptospirosis were included in this study. These samples have been described in detail previously [29]. Samples were collected between April 2009 and November 2013. Fifty-five acute samples were evaluated using MAT. Convalescent samples were not available for testing. The extraction of DNA was performed on-site in Brazil prior to the shipment of samples for testing. DNA was extracted using the DNeasy Blood & Tissue Kit (Qiagen, Germantown, MD) according to the manufacturer's recommendations. All extracted nucleic acids were stored at −80°C until use. Samples were tested using the UFI assay, pathogenic rtPCR, and the reference 16S rtPCR during a single freeze-thaw cycle. Fifty samples that tested positive in the UFI assay had amplicons were sequenced as described [29]; this included 47 samples that tested positive in the pathogenic rtPCR,

### Statistics

Assay comparisons were performed using two-tailed Fisher's exact tests. Comparisons of mean C_T_ values were performed using Welch t-tests. Fisher's exact tests and Welch t-tests were performed with GraphPad software (GraphPad; La Jolla, CA). Probit analysis was performed using SPSS (IBM; Armonk, NY).

## Results

### rtPCR Analytical Evaluation

**Figure 2:**
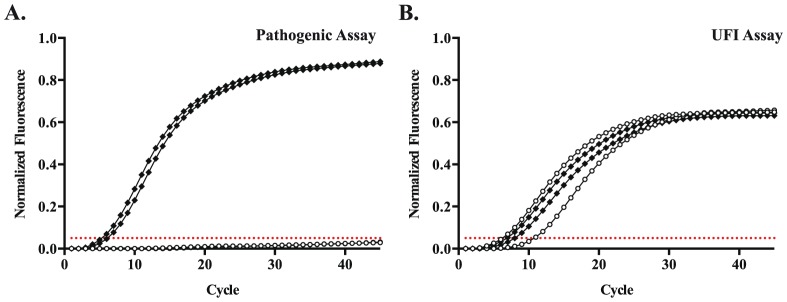
The pathogenic rtPCR does not amplify DNA from cultured *L. biflexa* isolates. Amplification curves for cultured isolates of *L. interrogans* (solid diamonds) and *L. biflexa* (open circles) in the (A) pathogenic rtPCR and (B) UFI assay. Results are displayed for two isolates of each species. The threshold for positivity is set at 0.05 normalized fluorescence units for both assays (dotted red line).

The dynamic range for the pathogenic rtPCR extended from 7.0 to 2.0 log_10_ copies/µL, and the 95% LLOD was 29 copies/µL. Extracted DNA from 39 cultured *Leptospira* isolates was tested (Table 1). Two non-pathogenic *L. biflexa* isolates produced no signal in the pathogenic assay (Figure 2A); these samples had C_T_ values of 6.26 and 10.80 in the UFI assay (Figure 2B). The remaining 37 isolates were detected with the pathogenic assay with C_T_ values from 2.81 to 11.88. No amplification was detected when extracted nucleic acids from 99 serum samples from Nicaraguan patients with dengue were tested. Extracted nucleic acids from Staphylococcal isolates and cultured strains of *S. enterica* subsp. *arizonae*, *S. enterica* serovar Typhi, *T. denticola*, and *B. burgdorferi* produced no amplification when tested in the pathogenic rtPCR.

The dynamic range of the reference 16S rtPCR extended from 5.0 to 1.0 log_10_ copies/µL. Extracted DNA from the 39 cultured *Leptospira* isolates were also evaluated using this assay. Thirty-five isolates were positive in the reference 16S rtPCR and demonstrated early C_T_ values (range 12.26–21.74). One strain each of *L. biflexa*, *L. weilii*, and *L. fainei*, tested positive but had late C_T_ values of 34.19, 34.48, and 36.03. The remaining *L. biflexa* isolate demonstrated no amplification.

### Clinical Samples

Available clinical data for the 65 cases of suspected leptospirosis is shown in Table 2. The day of illness (DOI) of sample collection was recorded for 45 patients; the median DOI was 8 (interquartile range 5–12). Of the 28 patients for whom the presenting signs and symptoms were available, the most common findings were fever (n = 26; 93%), myalgia (n = 19; 68%), and jaundice (n = 13; 46%).

**Table 2:** Reported clinical data for 65 samples from patients with suspected leptospirosis.

Patients, n	65
**Day of Illness**	
Patients, n (%)	45 (69.2)
Median (IQR)	8.0 (5–12)
**Clinical Presentation**	
Patients, n (%)	28 (43.1)
Fever	26
Myalgia	19
Jaundice	13
Gastrointestinal Complaints^1^	13
Headache	12
Respiratory Complaints^2^	8
Hemorrhage	8
Renal Failure	6
Conjunctival congestion	2

1 Includes nausea, vomiting, abdominal pain, diarrhea, anorexia, and hemorrhage.

2 Includes cough, shortness of breath, and hemoptysis.

All 65 samples were positive for *Leptospira* using the UFI assay, and no co-infections with dengue or malaria were detected (Table 3). Sixty-two (95.4%) samples were positive using the pathogenic rtPCR, and 24 (38.1%) samples were positive using the reference 16S rtPCR. The rates of *Leptospira* DNA detection using the UFI assay and pathogenic rtPCR did not differ significantly (p = 0.24). However, both assays detected *Leptospira* DNA in significantly more samples than the reference 16S rtPCR (p<0.0001 for both comparisons). The differences in rates of *Leptospira* detection using the reference 16S rtPCR (38.1%) and prior results using PCRs for *flaB* and *lipL41* (16/65 samples detected, 25.4%) did not reach statistical significance (p = 0.13).

**Table 3:** Pairwise comparisons of *Leptospira* PCR diagnostics.

		Pathogenic rtPCR		
		Positive	Negative	Total
**UFI Assay**	Positive	62	3	65
	Negative	0	0	0
**Reference 16S rtPCR**	Positive	24	0	24
	Negative	39	2	41
***flaB/lipL41***	Positive	15	1	16
	Negative	47	2	49
**Composite Reference**	Positive	28	1	29
	Negative	34	2	36

Comparison of the pathogenic *rt*PCR with the UFI assay, reference 16S rtPCR, conventional PCRs for *flaB/lipL41*, and a composite reference that takes into account *flaB/lipL41* and the reference 16S rtPCR. Samples that tested positive by at least one of these assays were considered positive, while those that tested negative by both assays were considered negative for this composite reference. Samples that tested negative in the pathogenic rtPCR had C_T_ values of 34.77, 35.26, and 36.11 in the UFI assay.

**Figure 3:**
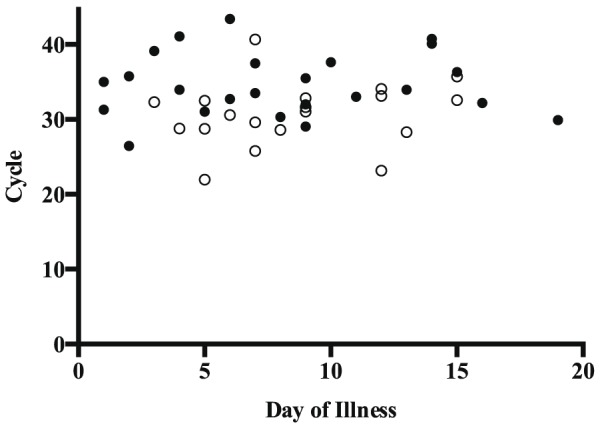
*Leptospira* DNA levels do not correlate with the day of illness of sample collection. The cycle threshold in the pathogenic rtPCR was compared to the day of illness of sample collection. Results are shown for the 43 samples detected in the pathogenic rtPCR with available day of illness information. Samples positive in the reference 16S rtPCR or conventional PCRs (open circles) and samples positive only in the UFI assay and pathogenic rtPCR (closed circles) are displayed.

For samples that only tested positive using the UFI assay and pathogenic rtPCR, the mean C_T_ value was significantly later (32.62; standard deviation, 2.21) than the mean C_T_ value for samples that also tested positive in the reference 16S rtPCR, 28.33 (standard deviation, 4.05; p<0.0001) (Table 4). When results in the pathogenic rtPCR were plotted against DOI of sample collection, no change in the C_T_ value was observed (Figure 3). MAT results were positive for 6 of 55 acute samples tested by this method. *Leptospira* DNA was only detectable in these six samples using the UFI assay and pathogenic rtPCR (Table 5).

**Table 4:** Amplicon sequencing results and C_T_ values for select clinical samples.

	n	Pathogenic rtPCR Positive, n (%)	UFI Assay Mean C_T_ (Standard Deviation)	Amplicons Sequenced	Sequence Matches^1^ (n)
**Reference 16S ** ***rt*** **PCR** **Positive** 1	24	24 (100)	28.33 (4.05)	13	*L. interrogans*, *L. kirschneri*, or *L. noguchii* (9)
					*L. borgpetersenii*, *L. santarosai*, or *L. weilii* (3)
					*L. meyeri, L. biflexa, or L. wobachii* (1)
***flaB/lipL41*** ** PCR and** **Reference 16S ** ***rt*** **PCR Negative**	36	34 (94.4)	32.62 (2.21)	34	*L. interrogans*, *L. kirschneri*, or *L. noguchii* (34)

1 Given the highly conserved nature of this region, final species determinations cannot be made from amplicon sequences.

**Table 5:** Amplicon sequencing and C_T_ values for select clinical samples evaluated by MAT.

	n	Pathogenic rtPCR Positive, n (%)	UFI Assay Mean C_T_ (Standard Deviation)	Amplicons Sequenced	Sequence Matches^1^ (n)
**MAT Positive**	6	6 (100)	32.51 (2.27)	6	*L. interrogans*, *L. kirschneri*, or *L. noguchii* (6)
**MAT Negative**	49	47 (95.9)	30.49 (3.90)	39	*L. interrogans*, *L. kirschneri*, or *L. noguchii* (35)
					*L. borgpetersenii*, *L. santarosai*, or *L. weilii* (3)
					*L. meyeri, L. biflexa, or L. wobachii* (1)

1 Given the highly conserved nature of this region, final species determinations cannot be made from amplicon sequences.

**Figure 4:**
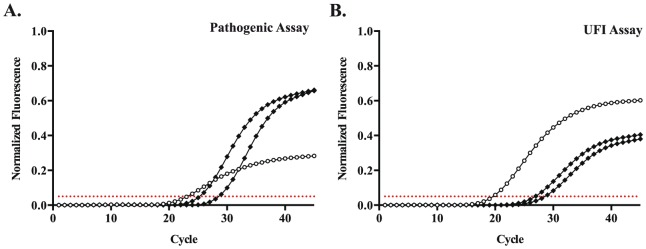
The pathogenic rtPCR shows blunted amplification of a clinical sample with 16S sequence consistent with a non-pathogenic *Leptospira* strain. Amplification curves for a clinical sample with sequence matching the non-pathogenic *Leptospira, L. meyeri*, *L. biflexa*, and *L. wolbachii* (open circles) in the (A) pathogenic rtPCR and (B) UFI assay. Two clinical samples with 16S sequence matching pathogenic strains (solid diamonds) are shown for comparison. The threshold for positivity is set at 0.05 normalized fluorescence units for both assays (dotted red line).

For 50 clinical samples, bidirectional sequence was obtained using the forward and reverse *Leptospira* primers. This included 34/36 samples that were only detected using the pathogenic rtPCR or UFI assay. Forty-nine sequences matched publicly available sequences from pathogenic *Leptospira* species (Table 4). A single sequence matched the 16S gene from non-pathogenic species: *L. meyeri*, *L. biflexa*, or *L. wobachii*. This sample was detected in all molecular tests. However in the pathogenic rtPCR, the amplification curve was flattened compared to curves from pathogenic species (Figure 4A) and compared to the shape of the curve when the same strain was amplified in the UFI assay (Figure 4B).

## Discussion

In this study, we describe the development of an rtPCR for the detection of pathogenic *Leptospira* species and present the results from a comparison of molecular diagnostics for *Leptospira* using samples from 65 suspected leptospirosis cases. The current study expands on earlier findings from our group by demonstrating the increased clinical sensitivity of the UFI assay as well as the pathogenic rtPCR when compared to another real-time nucleic acid amplification test, the reference 16S rtPCR.

The reference 16S rtPCR selected as a comparator in this study was originally reported by Smythe, et al., in 2002 [22], and primer and probe concentrations were later modified by Thaipadunpanit, et al. [8]. While these modifications may have affected the analytical performance of the assay compared to the original, this is unlikely to fully explain the difference in *Leptospira* detection rates observed here. The 16S rtPCR, as modified by Thaipadunpanit, et al., proved equally sensitive to MAT using acute and convalescent samples [8], [13]. In the current study, the reference 16S rtPCR demonstrated a similar dynamic range compared to the pathogenic rtPCR and results from 39 cultured isolates were consistent with published reports [8], [22], including the late amplification of some non-pathogenic strains.

Many nucleic acid amplification tests for leptospirosis have been developed that preferentially detect *Leptospira* species categorized as pathogenic [15], [18], [22], [28], [33]–[35]. While such assays may prove useful in other contexts, such as testing environmental samples [28], [36], [37], the results of this evaluation highlight concerns regarding such a testing approach for human specimens. Of the clinical samples that were successfully sequenced, 49/50 yielded sequence consistent with pathogenic *Leptospira* species. This includes 34/34 samples that were not detected using the reference 16S rtPCR or conventional PCRs for *flaB* and *lipL41*, and 3/3 samples that were negative in the pathogenic rtPCR. One sample most closely matched the 16S sequence of several *Leptospira* species considered non-pathogenic: *L. meyeri*, *L. biflexa*, and *L. wobachii*. However, it should be noted that some *Leptospira* strains originally identified as *L. meyeri* have been reclassified as pathogenic species [38]. This specimen was obtained from a patient who presented with fever, conjunctival congestion, vomiting and jaundice, and the sample tested positive in all assays evaluated. This case suggests that distinguishing pathogenic from non-pathogenic strains may not be clinically relevant in symptomatic patients with suspected leptospirosis. It also underscores the difficulty of designing an assay for pathogenic *Leptospira* species based on an evolving classification system. Interestingly, this sequence contained a single base difference compared to the consensus sequence of non-pathogenic strains, which resulted in an extra three bases that matched the 5′ end of the pathogenic probe. Such a difference may have allowed sufficient binding to generate a clear but blunted signal in the pathogenic rtPCR (Figure 4A), while even very high concentrations of reference isolates of *L. biflexa* produced no detectable signal (Figure 2A).

Leptospiremia occurs during the acute phase of clinical illness, though the duration remains poorly defined. It has been reported that nucleic acid amplification methods for the diagnosis of leptospirosis may only be useful during the first week of illness, though in untreated cases, *Leptospira* DNA has been detected past day 15 [2], [3], [8], [12], [26]. In a study by Agampodi, et al., the sensitivity of a 16S rtPCR was not affected by the length of time between the onset of symptoms and sample collection [26]. Consistent with that finding, we detected *Leptospira* DNA in samples collected up to 19 days after the reported onset of symptoms. Also, there was no apparent relationship between the DOI of sample collection and C_T_ values in the pathogenic rtPCR (Figure 3). This finding warrants further study, including an evaluation of serial samples from individual patients, as the duration of leptospiremia or the rate of change in bacterial load may be predictive of patient outcomes.

While the sample size was sufficient to demonstrate improved *Leptospira* detection using the UFI assay and pathogenic rtPCR, the number of clinical samples and available information were insufficient to evaluate correlations between disease severity and the level of leptospiremia. The current study also involved clinical samples from a single geographical location, and our findings will need to be confirmed in other regions. A limitation to the pathogenic rtPCR, which is common to all nucleic acid amplification tests, involves a concern regarding the emergence of divergent bacterial strains with mutations in the sequences targeted by the primers and probes. We have attempted to address this limitation by targeting highly conserved regions of available *Leptospira* sequences, but this concern cannot be eliminated.

In conclusion, we present the development of a pathogenic rtPCR for *Leptospira*. Using a set of 65 clinical samples, the pathogenic rtPCR as well as the UFI assay demonstrated significantly improved clinical sensitivity compared to the reference 16S rtPCR. Future studies are needed to investigate the clinical and epidemiologic significance of more sensitive *Leptospira* detection using these assays.
